# The Possibilities of Hereditary Transmissions of Oral Lesions

**Published:** 1884-09

**Authors:** W. C. Starbuck


### THE POSSIBILITIES OF HEREDITARY TRANSMISSIONS OF ORAL LESIONS.

BY W. C. STARBUCK, D. D. S.

Read at the Nineteenth Annual Meeting of the Massachusetts Dental Society, Boston, June 5th and 6th, 1884.

For years it has been my practice, in many cases, to remove the first permanent molar for the purpose of correcting a crowded and irregular condition of the teeth, and to prevent, as far as possible, approximal decay. I am well aware that there are many who most decidedly object to such a proceeding; but whatever may have been the experience of others in similar cases, I know from my own observations that great benefits have resulted by the timely removal of such teeth as in my judgment have seemed expedient, in order to obtain the best relief for existing conditions. This is a subject upon which a great deal has been and can be said, and which has not only agitated the minds of many of our best operators, but parties outside of the profession have expressed themselves as against the practice. I will mention one instance.

In looking over a book published a few years ago, entitled "The Life History of Our Planet," by William D. Gunning, I made the following extract, thinking that some time I might have occasion to refer to it:—

"By his *arm* man holds dominion over the world. Through his *mouth*, in a large sense, he is and was created.

"The dentist by thrusting his forceps in our mouths is fast making us over into a new race, a race which one day may take the name already coined for it, the name of 'lantern jawed.' It would seem our national malady is bad teeth. Examinations were made in the primary schools of a Boston suburb, which showed that out of three hundred boys and girls under the age of twelve, only fifteen had perfectly sound teeth! The dentist begins on little mouths, and his work will end by making a race with little mouths. If any one will examine a museum of skeleton heads collected years ago, as that of Dr. Morton, of Philadelphia, he will see that, generally, where teeth have been extracted the pits have not closed up. The explanation is that, in general, the teeth have been lost later in life. If now he were to examine such a museum as might be made out of living heads he would find that, generally, more teeth have been lost, and more of the alveola, or pits, had been closed up. When the jaw loses a tooth and is so young and vital as to mend the chasm, finding itself too large for its uses, it contracts. Suppose that early in life teeth have been extracted. The jaw, being too large for its needs, would tend to contract and lose its roundness of outline. It would become more triangular and pointed. It would assume the form meant to be described by that ill-chosen word, 'lantern jaw.'

"The contraction of the jaw becomes hereditary. It is the concurrent testimony of American dentists that our teeth, when sound and in their places, crowd each other for room. The jaw is too small. If nature meant it to carry sixteen teeth she has not the intelligence to measure space against number. And here the dentist comes to help her again. He pulls out one or more of her sound teeth to give room to the others—and to let her contract the jaw a little more."

The statement of Mr. Gunning that "the contraction of the jaw becomes hereditary," led me to see what some of the authorities had to say upon the subject. Ribot, in his work on Heredity says, "that nothing is more undisputed than the heredity of the form, size and anomalies of the osseous system; and universal, every-day experience, proves the heredity of all the proportions of the cranium, thorax, pelvis, vertebral column, and the smallest bones of the skeleton." Even the heredity of the excess or defect in the number of the teeth has been ascertained. (Lucas.)

I have among my patients several, where this peculiarity of defect in the number of teeth has been hereditarily transmitted. There is no way of proving, however, that these defects were owing to any external interference originally.

Anomalies accidentally acquired, according to Ribot, are transmissible, as are also artificial deformities.

Three tribes in Peru had each their own peculiar mode of deforming the heads of their children—and this deformity has since remained. It is said the Esquimaux cut off the tails of the dogs they harness to their sledges; the pups are often born tailless!

In considering the question of the possible transmission of accidental deformities and lesions, I will refer to a portion of the report of the proceedings of the Ohio State Dental Society, of 1882. The subject under discussion was "What shall be done with the first permanent molars?"

A member inquired if an abnormal condition of the mouth, produced by improper interference (meaning the extraction of these teeth), might be transmitted to offspring.

Dr. Taft replied that it had been claimed, with a good deal of plausibility, that at least some accidental deformities had been thus handed down.

Then Dr. Watt read the following note, entitled "Transmission of Traumatic Lesion."

Dear Sir: Mrs. B. when a girl about twelve years of age, had a fall, striking on a piece of broken crockeryware, which made a severe lacerated wound posteriorly and to the right of the mental process of the inferior maxillary bone, nearly penetrating the floor of the mouth. She married and became the mother of five children, all of whom have the duplication of cicatrix, and in the same place as the initial lesion possessed by the mother.

(Signed), W. Mitchell,
Delaware, O.

Dr. Watt remarked that he "regarded this as a very interesting item, and as dentists have much to do in causing 'traumatic lesions,' he thought it ought to be promptly and widely published, and added that if accidental deformities are ordinarily thus transmissible, we really incur heavy responsibilities in our manifold mutilations." He expresses the opinion, however, that extraction may become necessary to make room.

This is the very thing which I admit I have been doing, when necessary, for years, and in every case with the most gratifying result. I propose to show models of two mouths in illustration of the benefit of judicious extraction. One was given to me by a

Pennsylvania dentist—a model of his own mouth—where nine teeth have been extracted, eight molars, and one inferior incisior. He is a very small man, and he told me his teeth were large and in a crowded condition. It will be observed that the articulation of the remaining ones is good, and he said he had all the teeth he wanted or needed.

The other case in my own practice is that of a child, where the extraction of the first permanent molars was done in January and February, 1881, and the regulating commenced in March. Seven months after, the teeth were in position, as shown by the second model. After the regulation the appliances were removed, and there has been no apparent change of position up to the present time.

It has been said that in surgery we cut off a finger to save the others; we remove one eye to save the other. On the same principle why cannot we remove a tooth to correct an irregularity, or to save the others, without doing an incalculable injury to posterity, and without incurring the grave responsibilities referred to. We are all of us called upon daily to change the condition of things as we find them, and we should do it intelligently and conscientiously, according to our best light. In searching for justification for the course I have pursued in the past, and for encouragement in the future, I find the opinion expressed that accidental deformities are not usually transmitted, and that it takes "thousands of years" to change type. Ribot says that "deviations from a type, after having subsisted for generations, return to the normal state, so that many naturalists hold it as a rule that accidental modifications are *not* perpetuated." As far as I have been able to ascertain, neither Darwin nor Huxley has recorded any cases where the lesions of the oral cavity have been hereditarily transmitted. I shall therefore feel warranted in going on as before—not in performing "manifold mutilations"—but in doing all in my power to assist nature in her developments, relieving suffering humanity as circumstances arise, and leave the generations to come to combat with conditions as they find them; and, in the words of Marcus Antoninus, "If any man is able to convince me and show me that I do not think or act right, I will gladly change, for I seek the truth by which no man was ever injured. But he is injured who abides in his error and ignorance."

